# Bortezomib-Loaded Mesoporous Silica Nanoparticles Selectively Alter Metabolism and Induce Death in Multiple Myeloma Cells

**DOI:** 10.3390/cancers12092709

**Published:** 2020-09-21

**Authors:** Alessandra Nigro, Luca Frattaruolo, Mariarosa Fava, Ilaria De Napoli, Marianna Greco, Alessandra Comandè, Marzia De Santo, Michele Pellegrino, Elena Ricci, Francesca Giordano, Ida Perrotta, Antonella Leggio, Luigi Pasqua, Diego Sisci, Anna Rita Cappello, Catia Morelli

**Affiliations:** 1 Department of Pharmacy, Health and Nutritional Sciences, University of Calabria, 87036 Rende, Italy; nigroale16@gmail.com (A.N.); luca.frattaruolo@unical.it (L.F.); mariarosafava@live.it (M.F.); mariannagreco.89@gmail.com (M.G.); alessandracomande@outlook.it (A.C.); michele.pellegrino@unical.it (M.P.); riccielena91@gmail.com (E.R.); francesca.giordano@unical.it (F.G.); antonella.leggio@unical.it (A.L.); diego.sisci@unical.it (D.S.); 2 Department of Environmental Engineering, University of Calabria, 87036 Rende, Italy; ilariadenapoli8@gmail.com (I.D.N.); marziadesanto@gmail.com (M.D.S.); luigi.pasqua@unical.it (L.P.); 3 Department of Biology, Ecology and Earth Sciences, Centre for Microscopy and Microanalysis (CM2), Transmission Electron Microscopy Laboratory, University of Calabria, Rende, 87036 Cosenza, Italy; ida.perrotta@unical.it

**Keywords:** mesoporous silica nanoparticles, target therapy, bortezomib, multiple myeloma, apoptosis, energy metabolism, mitochondrial dysfunction, oxidative stress

## Abstract

**Simple Summary:**

Multiple myeloma (MM) is the second most common hematological malignancy and, despite the great advances made in its management, the development of novel therapeutic strategies are still needed in order to extend patients’ survival and to improve their quality of life. Here we show the striking ability of a mesoporous silica-based device to selectively deliver the antineoplastic drug bortezomib to Folate Receptor (FR) overexpressing MM cells, without causing injury nor perturbing the metabolic homeostasis of FR-negative healthy cells. Our data highlight the high efficacy and extraordinary safety of the tested nanodevice, paving the way for its future exploitation in the treatment of MM.

**Abstract:**

A mesoporous silica-based nanodevice bearing the antineoplastic drug bortezomib (BTZ), whose release is triggered in acidic environment and grafted with folic acid (FOL) as a targeting function (FOL-MSN-BTZ) was tested on folate receptor overexpressing (FR+) multiple myeloma (MM) cells and on FR negative (FR−) normal cells. FOL-MSN-BTZ efficacy studies were conducted by means of growth experiments, TEM, TUNEL assay and Western Blotting analysis (WB). Metabolic investigations were performed to assess cells metabolic response to MSNs treatments. FOL-MSN-BTZ exclusively killed FR+ MM cells, leading to an apoptotic rate that was comparable to that induced by free BTZ, and the effect was accompanied by metabolic dysfunction and oxidative stress. Importantly, FOL-MSN-BTZ treated FR− normal cells did not show any significant sign of injury or metabolic perturbation, while free BTZ was still highly toxic. Notably, the vehicle alone (MSN-FOL) did not affect any biological process in both tested cell models. These data show the striking specificity of FOL-MSN-BTZ toward FR+ tumor cells and the outstanding safety of the MSN-FOL vehicle, paving the way for a future exploitation of FOL-MSN-BTZ in MM target therapy.

## 1. Introduction

Multiple myeloma (MM) is a B-cell neoplasm characterized by uncontrolled growth of malignant plasma cells and monoclonal immunoglobulins secretion within the bone marrow. It is the second most common hematological malignancy, with a median incidence of 6/100,000 worldwide [1].

One of the main therapeutic strategies currently applied in MM clinical practice involves the use of proteasome inhibitors, a drug class for which bortezomib (BTZ) is the progenitor [2]. BTZ binds and inhibits the catalytic subunit of 26S proteasome complex, leading to the accumulation of misfolded proteins in endoplasmic reticulum (ER) lumen (ER stress phenomena) and reactive oxygen species (ROS). The concomitant accumulation of ubiquitinated proteins in the mitochondria, occurring after proteasome inhibition, leads to the impairment of cellular respiration, triggering the apoptotic death pathways in MM cells [3,4]. Clinically relevant side effects of BTZ, due to its low specificity towards cancer cells, include hematological toxicity, such as thrombocytopenia, and the bortezomib-induced peripheral neuropathy (BIPN), which occur in approximately 30–40% of patients and represent the main limit for BTZ use in MM treatment [5]. Indeed, at neuronal level, interference of BTZ with the ubiquitin-proteasome system leads to misfolded-protein accumulation, calcium homeostasis disruption, impairment of mitochondrial energy metabolism and oxidative stress, which ultimately cause axonal transport degeneration and loss of neuronal functionality [6].

Despite the great advances made in the management of MM during the last 20 years having led to progressively higher complete remission rates, the majority of patients will inevitably relapse; therefore, MM remains an incurable disease. The search for a cure has remained elusive and the development of novel therapeutic options, aimed at extending patients’ survival and at improving their quality of life, are still needed [1].

In this context, nanotechnology offers multiple benefits by serving as a means for the controlled and target-oriented delivery of precise molecules, preserving healthy tissues [7]. Among the platforms that are frequently considered in the design of target-specific nanostructured drug delivery systems (metallic, organic, inorganic and polymeric nanostructures), growing evidence reinforce the idea that mesoporous silica nanoparticles (MSNs) excellently fulfil all the requested criteria [8,9]. Silica is classified by FDA as “Generally Recognized as Safe” and has been used for a long time as a food additive, as well as in cosmetics and in pharmaceutical formulations [10,11,12]. Among the various silica nanoparticles proposed as DDSs, MSNs remain the safest choice [13]. This might depend on the fact that MSNs are degraded into smaller silica oligomers and water-soluble orthosilicic acid (Si(OH)_4_), which are easily excreted with urine. In addition, pore size, shape and surface features seem to have a great impact on the biodistribution and biocompatibility of MSNs [14]. Indeed, the safety and toxicity of nanoparticles represent one of the major concerns for scientists with respect to their further clinical translation, owing to their high surface-to-volume ratio compared to other counterparts [15].

In particular, MSNs offer a solid framework with a bifunctional surface, internal and external, making them a highly versatile tool, with regularly sized pores, in the range of 15–100 Å, which allow the encapsulation of a variety of hydrophobic and hydrophilic therapeutic agents, and a high surface area that is able to host even multiple target molecules on the same particle. These properties endow the MSNs with the unique advantage of directly delivering therapeutic/diagnostic (theranostic) agents (e.g., drug, siRNA, miRNA, enzymes, proteins, DNA, as well as probes for imaging applications) to the desired location (e.g., tumors) [16,17,18,19].

Here, we accurately describe the in vitro behavior of a MSU-type mesoporous silica nanosystem bearing the folic acid (FOL) as targeting function and loaded with the proteasome inhibitor BTZ, which is bound to the device [20]. The material system employed, whose design, development and ongoing in vivo validation will be included in a manuscript that is about to be submitted, is a multifunctional nanostructured device specifically recognized and internalized by cells. Once the targeted tissue has been reached, such pH-tunable devices are expected to efficiently release the drug at the low pH of tumor microenvironment, due to the enhanced glycolytic rate of cancer cells and the consequent increase in intracellular lactic acid [21].

Folic acid (FOL) is widely recognized to possess a targeting function that can be anchored on MSNs. Being an essential vitamin for DNA synthesis and cell replication, folic acid is particularly important for cancer cells that have a high replication rate. Indeed, several types of cancers, including MM, express high levels of Folate Receptor (FR) on their membranes, compared to lower or no expression in normal cells [22,23,24], and their density increases with the cancer grade [25]. Moreover, although FR is expressed in the epithelial cells of several normal tissues (e.g., lungs, ovary, uterus [26]), its luminal localization protects from FR-targeted folate conjugates administered intravenously [27]. These features make FRs interesting Tumor Associated Antigens (TAAs) to be exploited for targeting imaging molecules and therapeutic compounds directly to cancerous tissues [28]. In humans, three FR isoforms have been described, referred to as isoform α, β and γ, each with tissue-specific distribution [29]. The α and β isoforms are glycosylphosphatidylinositol (GPI)-anchored membrane proteins which can internalize folates and folate conjugated compounds via a receptor-mediated endocytosis [30]. In this context, we already reported that our FOL grafted MSNs (MSN-FOL) do not enter cells unless opportunely functionalized, and that a highly specific, receptor-mediated, cellular internalization of MSN-FOL occurs exclusively in FR expressing cells [9]. Therefore, FOL-MSN-BTZ devices should preferentially deliver the drug to FR-expressing target tissues, preserving the cargo from premature leakage and metabolism, thus reducing or even abolishing BTZ-induced side effects.

Here, FOL-MSN-BTZ and the vehicle alone (MSN-FOL) were investigated in vitro in terms of biocompatibility and efficacy on tumor and normal cell models, expressing or not FR, respectively. Moreover, the pro-oxidant functional groups on silica nanoparticles and their interactions with cell membranes could represent an important source of oxidative stress [31]. In fact, MSNs have been reported to inhibit cellular and mitochondrial respiration [32], to induce size-dependent genotoxicity and oxidative stress on endothelial cells [33] and to increase the intracellular ROS levels via mitochondrial membrane depolarization, impairment of the electron transport chain and activation of NADPH oxidases [32]. Therefore, an accurate analysis of the metabolic perturbations associated with MSNs treatment has been also performed, mainly in order to assess the safety of the vehicle, excluding the possibility of early metabolic and toxicological injuries that it might induce.

## 2. Results and Discussion

### 2.1. FOL-MSN-BTZ Selectively Kills FR Expressing Cells

The biocompatibility and efficacy of FOL-MSN-BTZ and vehicle alone (MSN-FOL) were first investigated on tumor and normal cell models, expressing FR or not.

**Figure 1:**
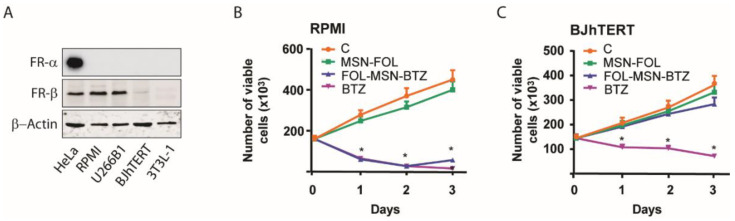
FOL-MSN-BTZ kills FR+ MM cells but not normal cells. (**A**) 50 μg of non-denatured proteins (for FR-α detection) and denatured proteins (for FR-β detection) from total lysates of indicated cell lines were loaded and subjected to WB analysis. β-actin was used as loading control. FOL-MSN-BTZ induces death in FR+ RPMI-8226 (RPMI) MM cells (**B**) but not in FR- BJhTERT normal cells (**C**). Cells were treated for 1h with FOL-MSN-BTZ or left untreated (C = control). MSN-FOL was used as negative control and the free drug BTZ as positive control. (*) *p* < 0.05 vs. control.

The effect of MSNs on cell proliferation was evaluated on human FRα-/FRβ+ MM cell lines, RPMI-8226, with BTZ being the treatment of choice for this type of cancer. The FRα-/FRβ- BJhTERT cell line was used as a normal cell model, not expressing FR (Figure 1A).

Strikingly, FOL-MSN-BTZ was able to selectively induce death only in FR+ RPMI-8226 cells (Figure 1B), but not in FR- BJhTERT normal cells (Figure 1C), while free BTZ was not selective and was toxic for both cell lines tested, independently of their FR expression (Figure 1B,C). Similar results were obtained in additional FR+ and FR- cell lines (Figure S1). Moreover, preliminary data from ongoing immunogold analysis, which will be included in a forthcoming manuscript, confirm the high selectivity of the device toward FR-expressing MM cells only.

Our observations clearly show that, when loaded into MSNs, BTZ loses its toxicity on normal cells. Last, but not least, it is worth mentioning that the vehicle per se (MSN-FOL) was not toxic to either normal or cancer cells (Figure 1B,C and Figure S1).

### 2.2. Drug-Loaded MSNs Trigger Apoptosis in MM Cells but not in Normal Cells

**Figure 2:**
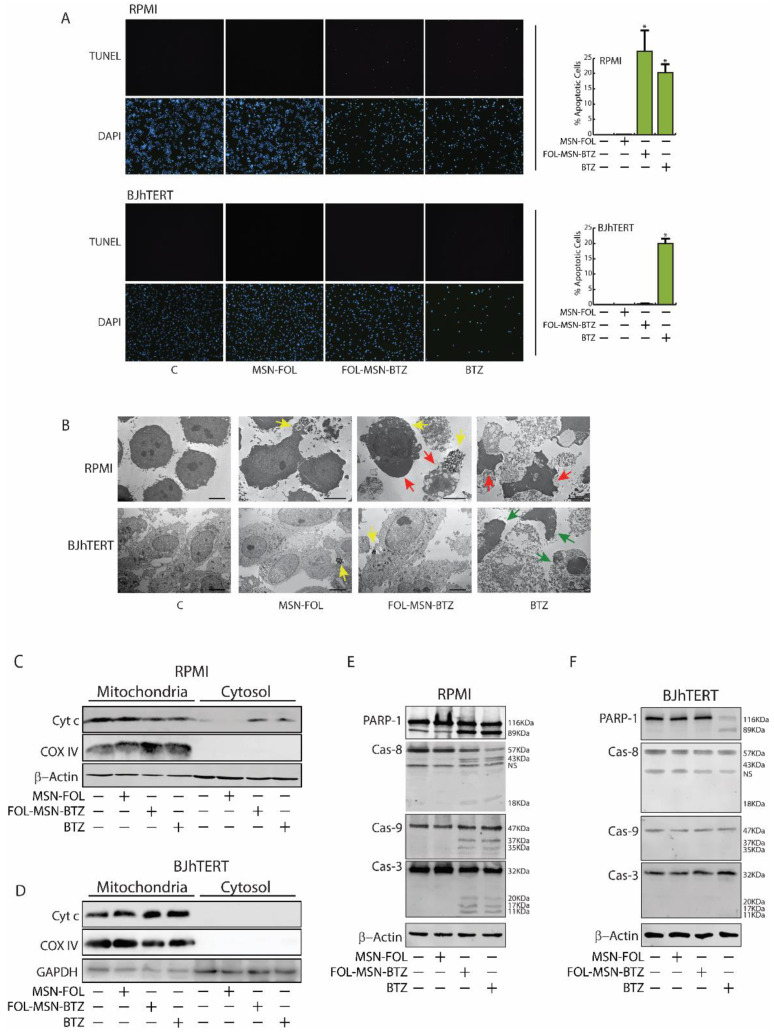
BTZ is not toxic to normal cells when bound to targeted MSNs. (**A**) RPMI-8226 (RPMI) and BJhTERT were treated or not (control) with MSN-FOL, FOL-MSN-BTZ and free BTZ for 1 h and processed for TUNEL assay after 36 h. Nuclei were counterstained with DAPI. Cells were photographed at 10× magnification, and apoptotic cells from triplicate experiments were counted using Image J software (graphs on the right). (*) *p* < 0.05 vs. control. (**B**) A duplicate set of cells was processed for TEM analysis (see *Materials and Methods*). Red arrows: apoptotic cells; green arrows: parthanatic cell death; yellow arrows: MSNs. Scale bars 5 μm, × 2000 magnification. (**C**–**F**) Molecular profile of BTZ-induced death in MM cells and in BJhTERT cells line. A third set of both cell lines was treated as in (**A**,**B**), and cytosolic and mitochondrial fractions (**C**,**D**) or total proteins (**E**,**F**) were extracted and subjected to WB analysis to assess the expression of the indicated apoptotic markers. β-Actin and GAPDH were used as loading control; COX IV: mitochondrial marker to assess fractionation quality. NS: Non-Specific bands.

BTZ anticancer activity occurs through multiple mechanisms. Proteasome inhibition increases the levels of pro-apoptotic proteins and decreases several anti-apoptotic proteins, triggering both the intrinsic (mitochondrial Cytochrome c release and Caspase-9 activation) and the extrinsic (Fas/Caspase-8-dependent) apoptotic pathways in malignant cells [34]. Moreover, recent evidence reports that the main mechanism of BTZ-induced cell death involves the accumulation of misfolded and non-functional proteins, normally degraded by the proteasome, as well as of ROS in the ER, leading to ER stress and DNA damage-induced apoptosis [35,36]. Therefore, in order to assess whether MSN-bound BTZ triggers the same death pathways induces by the drug alone, cell death analysis was conducted on MM and normal cells. Indeed, our results show that both FOL-MSN-BTZ and free BTZ lead to comparable apoptotic rates in FR+ MM RPMI-8226 treated cells (Figure 2A, upper panels), while negligible apoptosis was detected in FR- normal BJhTERT cells exposed to FOL-MSN-BTZ, confirming the striking specificity of MSN-bound BTZ towards tumor cells if compared to free BTZ (Figure 2A, lower panels).

TEM observations gave similar results. In Figure 2B (upper panels), the red arrows indicate apoptotic cells (characterized by intact plasma membrane and condensed cytoplasm and chromatin) in RPMI-8226 treated with both FOL-MSN-BTZ and free BTZ.

Accordingly, both treatments caused the translocation of Cytochrome c (Cyt c) from the mitochondria to the cytoplasm (Figure 2C), as well as the cleavage of caspases and of the nuclear enzyme poly(ADP-ribose)polymerase (PARP-1) (Figure 2E), an endogenous substrate of activated Caspase-3 [37]. The molecular profile shows that, as it occurs in BTZ-treated MM cells [38], FOL-MSN-BTZ also led to the concomitant activation of both the extrinsic (Caspase-8 dependent) and the intrinsic (Caspase-9-dependent) apoptotic pathway.

Interestingly, FOL-MSN-BTZ is not able to enter FR- BJhTERT (yellow arrows in Figure 2B show MSNs inside RPMI-8226 but outside BJhTERT cells), and therefore it did not cause any injury to these cells, while free BTZ seems to trigger parthanatos (Figure 2B, lower panels), a particular kind of death mediated by the over-activation of PARP-1 [39]. Indeed, the molecular analysis of BTZ treated normal cells did not reveal Cyt c translocation (Figure 2D), nor any active (cleaved) caspases (Figure 2F), both well-known hallmarks of apoptosis. Meanwhile, a cleaved PARP-1 (Figure 2F), cytoplasmic and nuclear condensation, plasma membrane disruption at later time points (Figure 2B, green arrows), and DNA fragmentation (Figure 2A, lower panels) might be consistent with parthanatos [40].

### 2.3. Effects of MSN-FOL and FOL-MSN-BTZ on Cellular Energetic Pathways

Energy pathways are highly sensitive to exposure to toxic agents; indeed, they can affect several intracellular biochemical processes (glycolysis, Krebs cycle, electron transport and OXPHOS), resulting in the abnormal production of ATP and in the release of heat and chemical byproducts into the extracellular environment [21].

Although our MSN-FOL, as vehicle alone, is well tolerated by different cellular systems (Ref. [9], Figure 1B,C and Figure 2), metabolic changes might still precede the effects on viability of treated cells. Therefore, in order to investigate whether early metabolic and toxicological injuries might be induced by MSN-FOL treatment and to compare the effects of FOL-MSN-BTZ on FR+ and FR- cell lines, an accurate metabolic analysis was performed on MM (FR+) and normal (FR-) cells.

Changes in glycolysis and mitochondrial respiration, the two major energy producing cellular pathways, were detected through real-time measurements of Extracellular Acidification Rate (ECAR) and of Oxygen Consumption Rate (OCR), respectively, by means of a Seahorse Extracellular Flux (XFe96) Analyzer.

**Figure 3:**
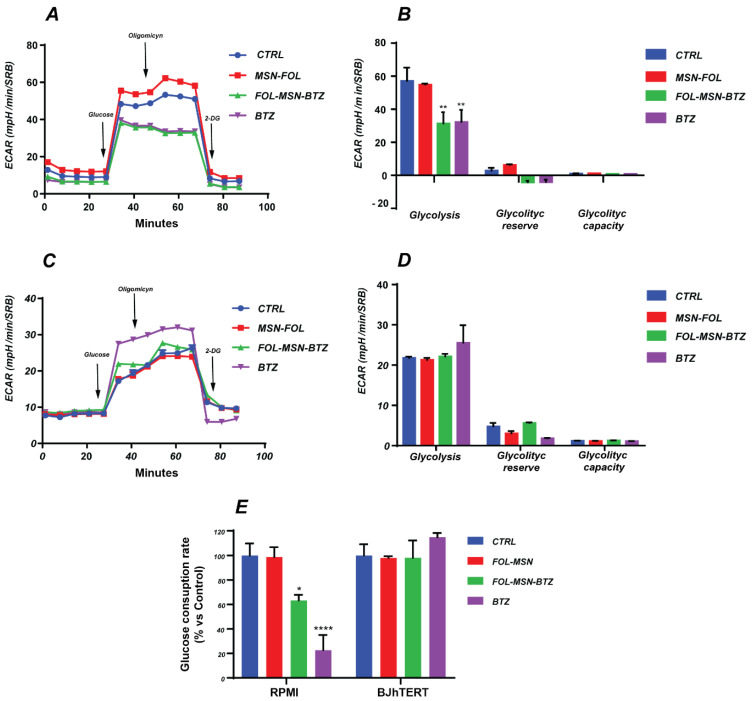
Glycolytic signature induced by BTZ-loaded MSNs in RPMI-8226 and BJhTERT cell lines compared to free BTZ. The glycolytic profile of cancer and normal cells treated for 1 h with FOL-MSN-BTZ, MSN-FOL and BTZ (as positive control) or left untreated (Control), was assessed 18 h after MSNs treatment using the Seahorse XF-e96 analyzer. The modulators glucose (30 min), Oligomycin (50 min) and 2-DG (70 min) were serially injected for ECAR measurement. A schematic tracing of ECAR flux in RPMI-8226 (**A**) and BJhTERT (**C**) and a graphic representation of Glycolysis, Glycolytic reserve and Glycolytic capacity in RPMI-8226 (**B**) and BJhTERT (**D**) are reported. (**E**) Glucose consumption was assessed in cancer and normal cells as described in the Section 3. Data represent the mean ± SEM of three independent experiments performed in sextuplicate and normalized by protein content (SRB). (*) *p* < 0.05; (**) *p* < 0.01; (****) *p* < 0.0001.

The Glycolysis stress test was performed by monitoring the ECAR variations following the consecutive additions of Glucose, Oligomycin and 2-deoxy-glucose (2-DG). Only FOL-MSN-BTZ, but not MSN-FOL, significantly affects the glycolytic metabolism of FR+ RPMI-8226 MM cells, with a trend that is perfectly comparable to that of the free drug (BTZ) (Figure 3A,B). In fact, both treatments led to a dramatic reduction of the glycolytic flux, with a significant decrease in the glucose oxidation process.

**Figure 4:**
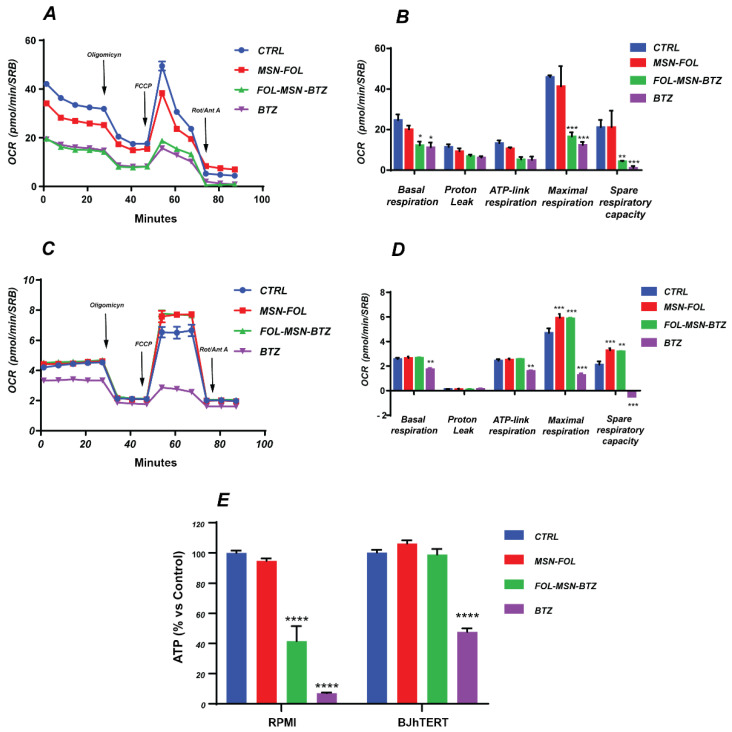
Mitochondrial signature induced by BTZ-loaded MSNs in RPMI-8226 and BJhTERT cell lines compared to the free BTZ. The mitochondrial profile of untreated (Control) cancer and normal cells or treated for 1 h with FOL-MSN-BTZ, MSN-FOL and BTZ (as positive control) was assessed 18 h after MSNs exposure using the Seahorse XF-e96 analyzer. Specific perturbations were serially injected at 30 min (Oligomycin), 50 min (FCCP) and 70 min (mix of rotenone and Antimycin A). A schematic tracing of OCR flux in RPMI-8226 (**A**) and BJhTERT (**C**) and graphic representation of the basal respiration, proton-leak, ATP-link respiration, maximal respiration and spare respiratory capacity in RPMI-8226 (**B**) and BJhTERT (**D**) are reported. (**E**) ATP profiling production in cancer and normal cells treated as in (A–D). Data are the mean ± SEM of three independent experiments performed in sextuplicate and normalized by protein content (SRB). (*) *p* < 0.05; (**) *p* < 0.01; (***) *p* < 0.001; (****) *p* < 0.0001.

On the other hand, MSNs (both vehicle alone or BTZ-bearing MSNs) did not have any effect on FR- BJhTERT cells, while free BTZ showed a slight tendency to stimulate glycolysis, although the increase was not significant (Figure 3C,D). This trend could reflect a compensatory response to the BTZ-induced impairment in the OXPHOS-driven ATP production (Figure 4 and Refs. [41,42]).

These data were confirmed in both cell lines by glucose consumption evaluation (Figure 3E). In particular, the impairment of the glycolytic metabolism was much more evident in BTZ compared to FOL-MSN-BTZ treated MM cells, very likely due to the slower “active” endocytosis process of the drug-loaded MSN system [9], compared to the faster, generally “passive”, diffusion across the plasma membrane of a free molecule [43].

To evaluate the effect of MSNs treatments on the different parameters of cellular respiration, the Mito Stress test was performed on MM and normal cells by monitoring the change in OCR following the addition of Oligomycin, FCCP and Rotenone/Antimycin A. It is important to premise that the OCR of RPMI-8226 cells, after an initial raise following the addition of FCCP, undergoes a rapid decay. The inability of RPMI-8226 cells to maintain high oxygen consumption following decoupling is characteristic of this cell line, and similar OCR trends have previously been reported [44,45].

The significant reduction of basal respiration, maximal respiration and spare respiratory capacity induced by both free BTZ and FOL-MSN-BTZ in MM cells (Figure 4A,B) confirms that BTZ drives mitochondrial dysfunction [46,47] and demonstrates once again that MSN-FOL-BTZ-induced cytotoxicity is a consequence of the same metabolic perturbation exerted by the free drug. Conversely, in normal FR- BJhTERT cells (Figure 4C,D), only the free drug significantly impairs mitochondrial respiration. Notably, MSN-FOL and FOL-MSN-BTZ seem to favor cell respiration by increasing the maximal respiration and spare respiratory capacity compared to the control (Figure 4D). Because FR expression is negligible in BJhTERT cells, we can only hypothesize that nonspecific MSNs–plasma membrane phospholipid interactions [48] in the extracellular compartment may trigger intracellular signals that could lead to an improvement in mitochondrial respiration. Plasma membrane caveolins, for their ability to interact with MSNs [9,49] and with mitochondria [50,51], might represent good candidates in mediating these MSNs’ nonspecific effects on cellular respiration, as an attempt to protect intracellular structures and activities from external environment perturbations.

However, it is worth underlining that MSN-FOL alone did not exhibit any significant effect on either the energy producing pathways in RPMI-8226 cells (Figure 3 and Figure 4) or in the overall glycolytic process in BJhTERT (Figure 3), confirming that the vehicle itself does not hamper cell metabolism.

The impairment in glycolysis and the OXPHOS-driven respiratory deficit observed in FOL-MSN-BTZ and BTZ-treated FR+ RPMI-8226 cancer cells fit well with the apoptotic cell death triggered by drug (Figure 2 and Ref. [4]). Strikingly, in normal cells, only free BTZ compromised the mitochondrial respiration, reflecting the low selectivity of the free drug, while mitotoxicity disappeared when BTZ was carried by MSNs. Since alterations of mitochondrial functionality have been described as the mechanism underlying BTZ-induced peripheral neuropathy (BIPN) [52,53], it is clear how MSNs-mediated delivery of BTZ has the potential to strongly reduce BTZ-induced side effects in normal, non-targeted, tissues.

Seahorse analysis results were confirmed by the evaluation of intracellular ATP levels (Figure 4E), which further highlighted the lack of toxicity of the vehicle alone (MSN-FOL) and the high selectivity of FOL-MSN-BTZ toward MM cells. Indeed, MSN-bound BTZ inhibited the ATP production exclusively in FR+ RPMI-8226, while free BTZ caused a significant ATP depletion in both FR+ and FR- cell lines (Figure 4E).

### 2.4. FOL-MSN-BTZ Selectively Impairs the Mitochondrial Function of FR+ MM Cells

**Figure 5:**
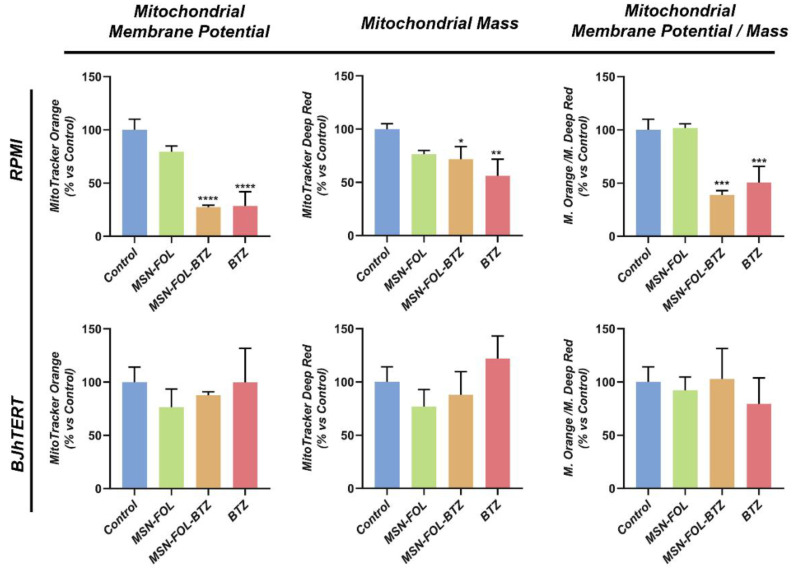
FOL-MSN-BTZ selectively affects mitochondrial function in MM cells. FR+ RPMI-8226 (RPMI) and FR- BJhTERT cells were treated or not (Control) with MSN-FOL, FOL-MSN-BTZ and free BTZ for 1 h and processed, after 24 h, for mitochondrial function analysis. Mitochondrial membrane potential and mitochondrial mass were assessed using MitoTracker Orange CM-H2TMRos and MitoTracker Deep Red probes, respectively, in RPMI and BJhTERT cells. The ratio between mitochondrial membrane potential and mitochondrial mass is also displayed. Values are expressed as mean ± SD of three independent experiments. (*) *p* < 0.05; (**) *p* < 0.01; (***) *p* < 0.001; (****) *p* < 0.0001.

The OCR decrease and the significant reduction in ATP levels prompted us to better elucidate the effect of free or MSN-bond BTZ on mitochondrial number and function. The MitoTracker Orange probe was used to monitor the effects of treatments on the electro-chemical gradient across the mitochondrial membrane, while MitoTracker Deep Red probe served to evaluate changes in the mitochondrial mass. In FR+ RPMI-8226 cells, a drastic reduction in mitochondrial membrane potential and in mitochondrial mass was observed following treatment with both free BTZ and BTZ-loaded MSNs, and the ratio of mitochondrial membrane potential/mass (index of functionality per mitochondrion) underlines that mitochondria are also altered in function (Figure 5). On the other hand, only a slight, but not significant, decrease of mitochondrial mass and potential was observed following treatment with MSN-FOL, a biological behavior consistent with the above-displayed Mito Stress test findings (Figure 4).

The same analysis, conducted under the same experimental conditions, did not show any significant effect on FR- normal BJhTERT cells (Figure 5).

### 2.5. FOL-MSN-BTZ Induces ROS Production in FR+ MM Cells Only

As mentioned, proteasome inhibition causes the accumulation of unfolded proteins, which triggers ER stress. The unresolved BTZ-induced ER stress seems to be the major mediator of BTZ cytotoxicity, priming cell death via multiple pathways, including ROS overproduction [54]. Oxidative stress can be mediated by Ca^2+^ leakage from the ER into the cytosol [54,55,56,57], thus leading to Cyt c release from the mitochondria and triggering apoptosis.

A significant contribution to cellular ROS levels is provided by cell energy metabolism, with superoxide anion (O_2_·^−^) representing the main byproduct of cellular respiration, generated by the incomplete reduction of O_2_ in the Electron Transport Chain (ETC) [58,59]. An impairment of cellular respiration with the concomitant mitochondrial accumulation of ubiquitinated proteins occur following proteasome inhibition and this metabolic alteration is responsible for an increase in mitochondrial ROS production at the ETC level [3].

Overall, calcium dysregulation and ROS production synergistically take part in the BTZ-induced apoptosis cascade [3] and are also involved in degeneration mechanisms of primary sensory neurons and intraepidermal nerve fibers that are responsible of BTZ-associated chronic peripheral neuropathy [60,61,62]. Therefore, a ROS detection assay was performed in tumor and normal cells in order to assess FOL-MSN-BTZ impact on their production, with respect to the free drug. The effect of MSN-FOL on cellular oxidative homeostasis was assessed as well.

**Figure 6:**
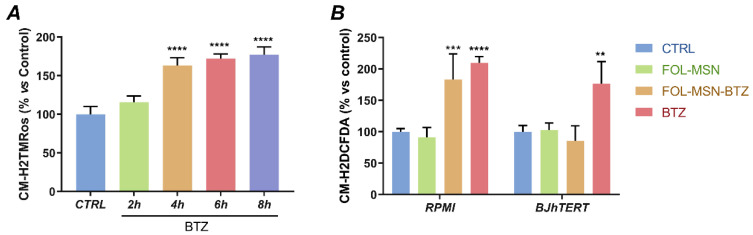
ROS species as mediators of BTZ-induced toxicity. (**A**) Time course assessment of ROS levels in FR+ RPMI-8226 (RPMI) cells treated with free BTZ (0.1 μg/mL). (**B**) Intracellular ROS levels were analyzed in RPMI MM cells and in FR- BJhTERT normal cells after treatment with MSN-FOL (negative control), FOL-MSN-BTZ and free BTZ (positive control). Untreated cells were used as negative control. Values represent the mean of three triplicate independent experiments. Data are shown as probe fluorescence normalized on viable cell number and expressed as percentage vs. negative control. (**) *p* < 0.01; (***) *p* < 0.001; (****) *p* < 0.0001.

Our results show that both FOL-MSN-BTZ and free BTZ lead to a comparable ROS production rate in FR+ RPMI-8226 treated cells, while a marked induction of ROS species was detected only in free BTZ treated FR- normal BJhTERT cells (Figure 6), confirming the striking specificity of MSN-bound BTZ towards tumor cells. Moreover, MSN-FOL does not trigger ROS production in any of the two cell lines tested, highlighting, once again, the safety of the mesoporous silica vehicle. These results corroborate the evidence assuming ROS involvement in the initiation of BTZ-associated apoptotic signaling. In fact, an increase in ROS generation induces Cyt c release from mitochondria with consequent caspases activation (Figure 2 and Refs. [63,64]).

## 3. Materials and Methods

### 3.1. Chemicals and Reagents

Dulbecco’s Modified Eagle’s Medium (DMEM), RPMI 1640 (1×) Medium, L-Glutamine, penicillin/streptomycin (pen/strep), Fetal Bovine Serum (FBS) and phosphate-buffered saline (PBS) were from Gibco™ (Life Technologies, Monza MB, Italy). Trypsin-EDTA solution 10×, protease inhibitors (cOmplete™ ULTRA Tablets, cat#5892970001), Digitonin (D141), formaldehyde and NP-40 were from MERCK/Sigma-Aldrich (Milan, Italy). Bortezomib was purchased from LC Laboratories (Woburn, MA, USA).

### 3.2. Cell Cultures and Treatments

Human FR positive (FR+) MM RPMI-8226 (RPMI) and U266B1 and cervix adenocarcinoma HeLa cell lines, as well as FR negative (FR−) embryonic kidney cells HEK293 [9] and normal mouse embryonic fibroblasts 3T3-L1 (Figure 1A) were purchased from ATCC, where they were authenticated. Cells were stored according to supplier’s instructions, and used within 6 months after frozen aliquot resuscitations. Normal foreskin fibroblast BJhTERT cells were kindly provided by Michael Lisanti, University of Salford, Manchester (UK). Cells were purchased from ATCC and transferred to our laboratory at passage *n* = 3 and handled as described above. RPMI and U266B-1 were cultured in RPMI-1640 medium, HeLa in Minimum Essential Medium (MEM) supplemented with non-essential amino acids, BJhTERT, HEK293 and 3T3-L1 in Dulbecco’s Modified Eagle’s Medium (DMEM). All media contained 10% FBS, 100 IU/mL pen/strep and 0.2 mM L-Glutamine. Mycoplasma negativity was tested monthly (PlasmoTest, Invivogen, Aurogene, Rome, Italy). For cell treatments, MSNs were resuspended in serum-free media (SFM) on a magnetic stirrer, and a ratio of 1 μg MSNs/10^5^ cells was used on the basis of titration experiments and added to the cells for 1 h. Free BTZ was added, as positive control, in the amount of 0.1 μg/mL, corresponding to the percentage of BTZ (10% of total MSNs weight) carried by FOL-MSN-BTZ.

### 3.3. Cell Proliferation Assays

MSNs’ effect on cell proliferation was assessed by trypan blue exclusion assay. Cells were seeded in triplicates for each condition, synchronized in SFM for 24 h and then treated for 1 h with 1 μg MSNs/10^5^ cells. Cells were then switched to fresh growth medium (GM) plus 1% FBS and counted after 72 h. Cell viability was determined by Countess^®^ II Automated Cell Counter (Invitrogen, Life Technology), according to supplier’s instructions.

### 3.4. TUNEL Assay

Apoptotic cells were determined by enzymatic labeling of DNA strand breaks using a Dead End Fluorimetric TUNEL System (Promega, Milan, Italy) according to the manufacturer’s instructions, in order to detect the DNA fragmentation, a hallmark of apoptosis. Briefly, 3 × 10^5^ cells were seeded on coverslips (Poly L-lysine-coated slides were used for RPMI-8226 cells to allow cell attachment) and treated as described for proliferation assays. After 36 h, coverslips were mounted on slides using Fluoromount mounting medium (MERCK/Sigma-Aldrich) and observed under a fluorescence microscope (Olympus BX51, Olympus Italia, Milan, Italy). DAPI was used to counterstain the nuclei. Apoptotic cells were photographed at 10× magnification, using ViewFinder™ 7.4.3 Software, through an Olympus camera system dp50 and then counted by Image J software (NIH, Bethesda, MD, USA).

### 3.5. Western Blotting (WB) Assay

Protein expression of FRs, Casp-3/8/9, PARP-1, Cyt C, COX IV and β-actin, were assessed by Western blotting (WB) using cytosol and mitochondrial protein lysates from RPMI-8226 and BJhTERT cell lines. Cells were plated in GM for 24 h and, after 1 h MSNs treatment, shifted to fresh GM medium. After 36 h, cells were lysed as previously described [65]. Briefly, harvested cells were incubated (10 min on ice) with cytoplasmic buffer containing 1 M sucrose, 1 M Hepes, 1 M KCl, 1 M MgCl2, 1 M EDTA, 1 M EGTA, Digitonin 10% and 200 mM PMSF. The cytoplasmic fraction was collected after centrifugation (13,000 rpm, 5 min, 4 °C). Intact mitochondria containing pellets were washed twice with PBS and incubated with mitochondrial buffer (1 M Tris-HCL pH 7.4, 1 M EDTA, 1 M EGTA, TRITON X-100 plus inhibitors). Mitochondrial fraction was collected after centrifugation (13,000 rpm, 10 min, 4 °C). Original Western blot can be found at Figure S2.

50 μg (cytosolic extract) and 10 μg (mitochondrial extract) of denatured proteins, and non-denatured for FRs detection, were run on an 11% and 13% polyacrylamide gel, respectively.

The following primary Abs were employed: FOLR1 (AF5646, R&D Systems Inc., Minneapolis, MN, USA); FOLR2 (PA545768, ThermoFisher Scientific); caspase-3 (8G10, #9665), caspase-8 (1C12, #9746), caspase-9 (#9502S), COX IV (3E11, #4850), Cytochrome C (D18C7, #11940), GAPDH (14C10, #2118) all from Cell Signaling, The Netherlands; β-Actin (AC-15, A1978) and PARP-1 (sc-7150) were from Santa Cruz Biotechnology, Inc. IRDye (LI-COR Corporate, NE, USA) were used as secondary Abs. Images were acquired with the Odyssey FC Imaging System (LI-COR Corporate).

### 3.6. Seahorse XFe96 Metabolic Profile Analysis

Cellular metabolic profile was assessed by Mito Stress test and Glycolysis Stress test, by monitoring extracellular acidification rates (ECAR) and real-time oxygen consumption rates (OCR), determined using the Seahorse Extracellular Flux (XFe- 96) analyzer (Seahorse Bioscience, Agilent Technologies, North Billerica, MA, USA). Briefly, 40,000 cells/well were seeded, in a final volume of 125 μL, into XFe-96 96-well cell culture plates for 24 h. For non-adherent RPMI-8226 cells, the Seahorse microplates were coated with 50 μg/mL Poly L lysine (Sigma Aldrich) before seeding to allow cell attachment. For Glycolysis Stress test, cells were washed in XF assay media, maintained in 175 μL/well of XF assay media at 37 °C, in a non-CO_2_ incubator for 1 h and then processed. Glycolytic parameters calculation was obtained by sequential injections of 25 μL 80 mM glucose (glycolysis substrate), 9 μM Oligomycin (ATP-synthase inhibitor, which switches cell metabolism towards glycolysis, by blocking mitochondrial ATP production) and 1 M 2-deoxyglucose (2-DG) (glucose analogue, which competitively inhibits the glycolytic process).

For Mito Stress test, cells were washed in XF assay media supplemented with 10 mM glucose, 1 mM Pyruvate, 2 mM L-glutamine and adjusted at 7.4 pH, maintained in 175 μL/well of XF assay media at 37 °C, in a non-CO_2_ incubator for 1 h and then processed. Cellular respiration parameters calculation was obtained by sequential injections of 10 μM Oligomycin (inhibiting ATP synthase and reducing OCR), 9 μM FCCP (which uncouples oxygen consumption from ATP production, raising OCR to a maximal value) and 10 μM Rotenone/10 μM Antimycin A mix (which target complex I and complex III of electron transport chain, respectively, reducing OCR to a minimal value). The data set was analyzed using XFe-96 software, after the measurements had been normalized by protein content (Sulforhodamine B, SRB). All experiments were performed three times independently.

### 3.7. Sulforhodamine B (SRB)-Based In Vitro Toxicology Assay Kit

After Seahorse analysis, cells were washed, fixed, and stained with the SRB dye (Acid Red 52) (Sigma Aldrich). The incorporated dye was then released from the cells with a TRIS base solution. An increase or decrease in the number of cells (total biomass) results in a concomitant change in the amount of dye incorporated by the cells in the culture. The signal was detected spectrophotometrically, measuring absorbance at a wavelength of 565 nm.

### 3.8. ROS Detection

Total intracellular ROS were quantified as previously reported [66]. Briefly, cells were plated and left in GM for 24 h and then treated for 1h with MSNs. After 6 h, cells were collected and the pellet was incubated in PBS containing 5 μM dye chloromethyl-2′,7′–dichlorofluorescein diacetate (CM-H2DCFDA, Molecular Probe, Invitrogen) in a humidified chamber at 37 °C for 1 h. Cells were then returned to GM and incubated at 37 °C for 20 min. The assay is based on the premise that the nonpolar, nonionic DCFHDA crosses cell membranes and is enzymatically hydrolyzed by intracellular esterases to non-fluorescent DCFH. In presence of ROS, DCFH is rapidly oxidized to highly fluorescent 2′,7′-dichlorofluorescein (DCF), detectable by a fluorescence microplate reader (Synergy H1 Hybrid Multi-Mode Reader, BioTek’s Hybrid Technology, Milan, Italy) in an excitation/emission range of 492–495/517–527 nm. Probe fluorescence in each sample is normalized on viable cells, counted by means of Countess^®^ II Automated Cell Counter (Invitrogen, Life Technology).

### 3.9. Evaluation of Mitochondrial Mass and Mitochondrial Membrane Potential

Mitochondrial mass and membrane potential were measured by FACS analysis of cells stained with MitoTracker^®^ Deep Red (mitochondrial mass evaluation) or MitoTracker^®^ Orange CM-H2TMRos (mitochondrial membrane potential evaluation) (Life Technologies), respectively, as previously reported [67]. Briefly, cells were plated and left in GM for 24 h, treated or not for 1 h with MSNs or free BTZ. After 24 h, cells were collected and incubated with MitoTracker staining solution (10 nM final concentration in PBS) for 30–60 min at 37 °C. Cells were then harvested, re-suspended in PBS and analyzed by flow cytometry (CytoFLEX Beckman, Beckman Coulter, Milan, Italy). Data analysis was performed using CytExpert Beckman Coulter software (Beckman Coulter, Milan, Italy).

### 3.10. Quantitation of Cellular ATP Levels

Intracellular ATP levels have been assessed by means of The CellTiter-Glo^®^ Luminescent Cell Viability Assay (Promega, Milan, Italy). Cells were plated in opaque-walled 96-multiwell plates, in GM and left to grow overnight. Next day, cells were treated or not with MSNs and free BTZ for 1 h. The next day, CellTiter-Glo^®^ Reagent was added to GM (1:1). Plates were incubated for 2 min on an orbital shaker to induce cell lysis. The luminescent signal intensity was detected by using Synergy H1 Hybrid Multi-Mode Reader (BioTek Hybrid Technology, Milan, Italy). The luminescence signal in each sample was normalized to protein content revealed by SRB assay.

### 3.11. Determination of Cellular Glucose Consumption

The measurement of extracellular glucose consumption was carried out by The Glucose-Glo™ Assay that couples glucose oxidation and NADH production with a bioluminescent NADH detection system. Cells were plated in opaque-walled 96-multiwell plates in GM, where they were left to grow overnight. On the next day, the cells were treated or not with MSNs and free BTZ for 1 h. 24 h after treatments, 50 μL of diluted GM (1: 200 in PBS) from each well were added to an equal volume of Glucose Detection Reagent, prepared according to manufacturer instructions. After 60 min of incubation at RT, plates were mixed for 30–60 s and the luminescent signal was read by means of the Synergy H1 Hybrid Multi-Mode Reader. The luminescence signal in each sample was normalized to cellular protein content revealed by SRB assay.

### 3.12. Statistical Analysis

Statistical analysis was performed using Student’s *t* test and ANOVA. Data were analyzed using GraphPad Prism 8 (GraphPad Software, Inc., San Diego, CA, USA) and reported as the mean ± SD of at least 3 independent experiments, each performed in triplicates. * *p* ≤ 0.05 vs. control.

## 4. Conclusions

In recent years, many agents and therapeutic combinations have been approved with the aim of achieving new possible treatment options in the management of MM [68]. However, despite the great progress made in the field, the current treatments are not fully satisfactory in terms of efficacy and tolerance; therefore, the development of novel therapeutic alternatives is still needed. Here we describe the biological behavior of a MSN-based nanodevice (FOL-MSN-BTZ) [20] able to selectively deliver BTZ to MM cells in a highly FR receptor-specific manner, sparing FR not-expressing normal cells.

Once inside the cells, the more acidic environment triggers BTZ release, inducing apoptosis and impairing glycolysis and OXPHOS-driven mitochondrial respiration, also nourished by a strong ROS production. On the other hand, FOL-MSN-BTZ does not enter FR− normal cells, and since no cellular damage or metabolic alterations are observed, we can conclude that no drug leakage from the particles occurs outside the cells, underlining the great stability of the FOL-MSN-BTZ nanosystem.

**Figure 7:**
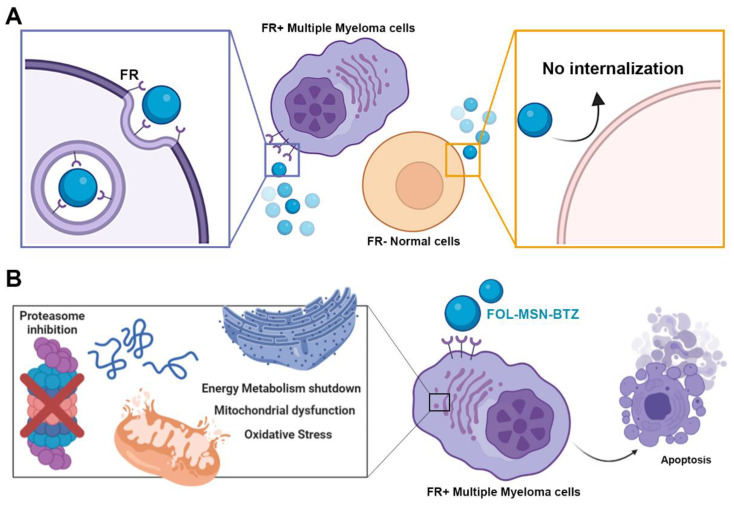
FOL-MSN-BTZ selectively alters metabolism and induce death in FR+ MM cells. (**A**) FOL-MSN-BTZ is selectively internalized by FR+ MM cells, but not by FR− normal cells. (**B**) Once inside the target cell, FOL-MSN-BTZ inhibits the proteasome machinery, which, in turn, leads to metabolic dysfunction and oxidative stress, eventually triggering apoptosis.

Importantly, the vehicle itself (MSN-FOL) not only does not affect the activity of the loaded drug toward cancer cells, but is also extremely safe for both internalizing tumor cells and not internalizing healthy ones. Indeed, MSNs–cells interaction does not even alter cellular metabolism, which is one of the earliest sensing pathways to be perturbed by noxious external (or internal) stimuli, confirming the high biocompatibility of our systems. Figure 7 shows a schematic representation of the proposed device and its selectivity towards MM FR-expressing cancer cells.

It is worth underlining that the RPMI-8226 and U266B1 cell lines used in this study, although they are the most commonly used as MM models, do come from plasmacytoma or plasma cell leukemia, and are not always analogous to primary human MM cells [69]; thus, these findings will need to be confirmed in patients’ derived fresh MM.

The evidence gathered here, together with ongoing in vivo studies that are giving very encouraging results that will be included in a forthcoming article, suggest that FOL-MSN-BTZ represents a great opportunity for the targeted therapy of MM, being highly selective toward tumor cells while preserving healthy cells. Our data give crucial information that is mandatory for paving the way for the future exploitation of MSNs strategies in a clinical setting in the cure of MM.

## Supplementary Materials

The following are available online at https://www.mdpi.com/2072-6694/12/9/2709/s1, Figure S1: Shows additional MSNs treated FR+ and FR− cell lines; Figure S2 collects all the original WBs used in Figure 2.

## References

[1] Rajkumar S.V. (2020). Multiple myeloma: 2020 update on diagnosis, risk-stratification and management. Am. J. Hematol..

[2] Richardson P.G., Hideshima T., Anderson K.C. (2003). Bortezomib (PS-341): A novel, first-in-class proteasome inhibitor for the treatment of multiple myeloma and other cancers. Cancer Control.

[3] Maharjan S., Oku M., Tsuda M., Hoseki J., Sakai Y. (2014). Mitochondrial impairment triggers cytosolic oxidative stress and cell death following proteasome inhibition. Sci. Rep..

[4] Bonvini P., Zorzi E., Basso G., Rosolen A. (2007). Bortezomib-mediated 26S proteasome inhibition causes cell-cycle arrest and induces apoptosis in CD-30+ anaplastic large cell lymphoma. Leukemia.

[5] Field-Smith A., Morgan G.J., Davies F.E. (2006). Bortezomib (Velcade™) in the treatment of multiple myeloma. Ther. Clin. Risk Manag..

[6] Meregalli C. (2015). An overview of bortezomib-induced neurotoxicity. Toxics.

[7] Singh A.P., Biswas A., Shukla A., Maiti P. (2019). Targeted therapy in chronic diseases using nanomaterial-based drug delivery vehicles. Signal Transduct. Target. Ther..

[8] Jafari S., Derakhshankhah H., Alaei L., Fattahi A., Varnamkhasti B.S., Saboury A.A. (2019). Mesoporous silica nanoparticles for therapeutic/diagnostic applications. Biomed. Pharmacother..

[9] Morelli C., Maris P., Sisci D., Perrotta E., Brunelli E., Perrotta I., Panno M.L., Tagarelli A., Versace C., Casula M.F. (2011). PEG-templated mesoporous silica nanoparticles exclusively target cancer cells. Nanoscale.

[10] Health U.D.O., Services H. (2019). Food and Drug Administration CFR—Code of Federal Regulations Title 21.

[11] Nigro A., Pellegrino M., Greco M., Comandè A., Sisci D., Pasqua L., Leggio A., Morelli C. (2018). Dealing with skin and blood-brain barriers: The unconventional challenges of mesoporous silica nanoparticles. Pharmaceutics.

[12] Pasqua L., Leggio A., Sisci D., Ando S., Morelli C. (2016). Mesoporous Silica Nanoparticles in Cancer Therapy: Relevance of the Targeting Function. Mini-Rev. Med. Chem..

[13] Croissant J.G., Fatieiev Y., Khashab N.M. (2017). Degradability and Clearance of Silicon, Organosilica, Silsesquioxane, Silica Mixed Oxide, and Mesoporous Silica Nanoparticles. Adv. Mater..

[14] Croissant J.G., Fatieiev Y., Almalik A., Khashab N.M. (2018). Mesoporous Silica and Organosilica Nanoparticles: Physical Chemistry, Biosafety, Delivery Strategies, and Biomedical Applications. Adv. Healthc. Mater..

[15] Narayan R., Nayak U.Y., Raichur A.M., Garg S. (2018). Mesoporous silica nanoparticles: A comprehensive review on synthesis and recent advances. Pharmaceutics.

[16] Zhou Y., Quan G., Wu Q., Zhang X., Niu B., Wu B., Huang Y., Pan X., Wu C. (2018). Mesoporous silica nanoparticles for drug and gene delivery. Acta Pharm. Sin. B.

[17] Cha B.G., Kim J. (2019). Functional mesoporous silica nanoparticles for bio-imaging applications. Wiley Interdiscip. Rev. Nanomed. Nanobiotechnol..

[18] Nakamura T., Sugihara F., Matsushita H., Yoshioka Y., Mizukami S., Kikuchi K. (2015). Mesoporous silica nanoparticles for 19 F magnetic resonance imaging, fluorescence imaging, and drug delivery. Chem. Sci..

[19] Slita A., Egorova A., Casals E., Kiselev A., Rosenholm J.M. (2018). Characterization of modified mesoporous silica nanoparticles as vectors for siRNA delivery. Asian J. Pharm. Sci..

[20] Pasqua L., Leggio A., Liguori A., Morelli C., Andò S. (2019). EP3288955 (B1)-Bortezomib-Based Delivery System. https://patents.google.com/patent/WO2016174693A1/nl.

[21] Burns J.S., Manda G. (2017). Metabolic Pathways of the Warburg Effect in Health and Disease: Perspectives of Choice, Chain or Chance. Int. J. Mol. Sci..

[22] Zhou Y., Unno K., Hyjek E., Liu H., Zimmerman T., Karmakar S., Putt K.S., Shen J.Y., Low P.S., Wickrema A. (2018). Expression of functional folate receptors in multiple myeloma. Leuk. Lymphoma.

[23] Zagorac I., Loncar B., Dmitrovic B., Kralik K., Kovacevic A. (2020). Correlation of folate receptor alpha expression with clinicopathological parameters and outcome in triple negative breast cancer. Ann. Diagn. Pathol..

[24] Assaraf Y.G., Leamon C.P., Reddy J.A. (2014). The folate receptor as a rational therapeutic target for personalized cancer treatment. Drug Resist. Updates.

[25] Lu Y., Low P.S. (2002). Folate-mediated delivery of macromolecular anticancer therapeutic agents. Adv. Drug Deliv. Rev..

[26] Elnakat H., Ratnam M. (2004). Distribution, functionality and gene regulation of folate receptor isoforms: Implications in targeted therapy. Adv. Drug Deliv. Rev..

[27] Yi Y.S. (2016). Folate Receptor-Targeted Diagnostics and Therapeutics for Inflammatory Diseases. Immune Netw..

[28] Keasberry N., Yapp C., Idris A. (2017). Mesoporous silica nanoparticles as a carrier platform for intracellular delivery of nucleic acids. Biochemistry.

[29] Cha W., Fan R., Miao Y., Zhou Y., Qin C., Shan X., Wan X., Li J. (2017). Mesoporous silica nanoparticles as carriers for intracellular delivery of nucleic acids and subsequent therapeutic applications. Molecules.

[30] Sun X., Wang N., Yang L.-Y., Ouyang X.-K., Huang F. (2019). Folic acid and PEI modified mesoporous silica for targeted delivery of curcumin. Pharmaceutics.

[31] Abdal Dayem A., Hossain M.K., Lee S.B., Kim K., Saha S.K., Yang G.-M., Choi H.Y., Cho S.-G. (2017). The role of reactive oxygen species (ROS) in the biological activities of metallic nanoparticles. Int. J. Mol. Sci..

[32] Hozayen W.G., Mahmoud A.M., Desouky E.M., El-Nahass E.-S., Soliman H.A., Farghali A.A. (2019). Cardiac and pulmonary toxicity of mesoporous silica nanoparticles is associated with excessive ROS production and redox imbalance in Wistar rats. Biomed. Pharmacother..

[33] Zhou F., Liao F., Chen L., Liu Y., Wang W., Feng S. (2019). The size-dependent genotoxicity and oxidative stress of silica nanoparticles on endothelial cells. Environ. Sci. Pollut. Res..

[34] Llopis-Lorente A., García-Fernández A., Murillo-Cremaes N., Hortelão A.C., Patiño T., Villalonga R., Sancenón F., Martínez-Máñez R., Sánchez S. (2019). Enzyme-Powered Gated Mesoporous Silica Nanomotors for On-Command Intracellular Payload Delivery. ACS Nano.

[35] Llopis-Lorente A., García-Fernández A., Lucena-Sánchez E., Díez P., Sancenón F., Villalonga R., Wilson D.A., Martínez-Máñez R. (2019). Stimulus-responsive nanomotors based on gated enzyme-powered Janus Au–mesoporous silica nanoparticles for enhanced cargo delivery. Chem. Commun..

[36] Shen J., Putt K.S., Visscher D.W., Murphy L., Cohen C., Singhal S., Sandusky G., Feng Y., Dimitrov D.S., Low P.S. (2015). Assessment of folate receptor-β expression in human neoplastic tissues. Oncotarget.

[37] Thornberry N.A., Lazebnik Y. (1998). Caspases: Enemies within. Science.

[38] Mitsiades N., Mitsiades C.S., Poulaki V., Chauhan D., Fanourakis G., Gu X., Bailey C., Joseph M., Libermann T.A., Treon S.P. (2002). Molecular sequelae of proteasome inhibition in human multiple myeloma cells. Proc. Natl. Acad. Sci. USA.

[39] Fatokun A.A., Dawson V.L., Dawson T.M. (2014). Parthanatos: Mitochondrial-linked mechanisms and therapeutic opportunities. Br. J. Pharmacol..

[40] Jouan-Lanhouet S., Arshad M.I., Piquet-Pellorce C., Martin-Chouly C., Le Moigne-Muller G., Van Herreweghe F., Takahashi N., Sergent O., Lagadic-Gossmann D., Vandenabeele P. (2012). TRAIL induces necroptosis involving RIPK1/RIPK3-dependent PARP-1 activation. Cell Death Differ..

[41] Vangapandu H.V., Alston B., Morse J., Ayres M.L., Wierda W.G., Keating M.J., Marszalek J.R., Gandhi V. (2018). Biological and metabolic effects of IACS-010759, an OxPhos inhibitor, on chronic lymphocytic leukemia cells. Oncotarget.

[42] Liemburg-Apers D.C., Schirris T.J., Russel F.G., Willems P.H., Koopman W.J. (2015). Mitoenergetic Dysfunction Triggers a Rapid Compensatory Increase in Steady-State Glucose Flux. Biophys. J..

[43] Cocucci E., Kim J.Y., Bai Y., Pabla N. (2017). Role of Passive Diffusion, Transporters, and Membrane Trafficking-Mediated Processes in Cellular Drug Transport. Clin. Pharmacol. Ther..

[44] Fan S., Price T., Huang W., Plue M., Warren J., Sundaramoorthy P., Paul B., Feinberg D., MacIver N., Chao N. (2020). PINK1-Dependent Mitophagy Regulates the Migration and Homing of Multiple Myeloma Cells via the MOB1B-Mediated Hippo-YAP/TAZ Pathway. Adv. Sci..

[45] Dalva-Aydemir S., Bajpai R., Martinez M., Adekola K.U., Kandela I., Wei C., Singhal S., Koblinski J.E., Raje N.S., Rosen S.T. (2015). Targeting the metabolic plasticity of multiple myeloma with FDA-approved ritonavir and metformin. Clin. Cancer Res..

[46] Yin Y., Qi X., Qiao Y., Liu H., Yan Z., Li H., Liu Z. (2019). The Association of Neuronal Stress with Activating Transcription Factor 3 in Dorsal Root Ganglion of in vivo and in vitro Models of Bortezomib- Induced Neuropathy. Curr. Cancer Drug Targets.

[47] Tibullo D., Giallongo C., Romano A., Vicario N., Barbato A., Puglisi F., Parenti R., Amorini A.M., Wissam Saab M., Tavazzi B. (2020). Mitochondrial Functions, Energy Metabolism and Protein Glycosylation are Interconnected Processes Mediating Resistance to Bortezomib in Multiple Myeloma Cells. Biomolecules.

[48] Kettiger H., Quebatte G., Perrone B., Huwyler J. (2016). Interactions between silica nanoparticles and phospholipid membranes. Biochim. Biophys. Acta Biomembr..

[49] Ekkapongpisit M., Giovia A., Follo C., Caputo G., Isidoro C. (2012). Biocompatibility, endocytosis, and intracellular trafficking of mesoporous silica and polystyrene nanoparticles in ovarian cancer cells: Effects of size and surface charge groups. Int. J. Nanomed..

[50] Fridolfsson H.N., Kawaraguchi Y., Ali S.S., Panneerselvam M., Niesman I.R., Finley J.C., Kellerhals S.E., Migita M.Y., Okada H., Moreno A.L. (2012). Mitochondria-localized caveolin in adaptation to cellular stress and injury. FASEB J..

[51] Fridolfsson H.N., Roth D.M., Insel P.A., Patel H.H. (2014). Regulation of intracellular signaling and function by caveolin. FASEB J..

[52] Colvin L.A. (2019). Chemotherapy-induced peripheral neuropathy: Where are we now?. Pain.

[53] Ludman T., Melemedjian O.K. (2019). Bortezomib-induced aerobic glycolysis contributes to chemotherapy-induced painful peripheral neuropathy. Mol. Pain.

[54] Lipchick B.C., Fink E.E., Nikiforov M.A. (2016). Oxidative stress and proteasome inhibitors in multiple myeloma. Pharmacol. Res..

[55] Guang M.H.Z., Kavanagh E.L., Dunne L.P., Dowling P., Zhang L., Lindsay S., Bazou D., Goh C.Y., Hanley C., Bianchi G. (2019). Targeting Proteotoxic Stress in Cancer: A Review of the Role that Protein Quality Control Pathways Play in Oncogenesis. Cancers.

[56] El Arfani C., De Veirman K., Maes K., De Bruyne E., Menu E. (2018). Metabolic Features of Multiple Myeloma. Int. J. Mol. Sci..

[57] Zheng Z., Fan S., Zheng J., Huang W., Gasparetto C., Chao N.J., Hu J., Kang Y. (2018). Inhibition of thioredoxin activates mitophagy and overcomes adaptive bortezomib resistance in multiple myeloma. J. Hematol. Oncol..

[58] De Santis M.C., Porporato P.E., Martini M., Morandi A. (2018). Signaling Pathways Regulating Redox Balance in Cancer Metabolism. Front. Oncol..

[59] Cannino G., Ciscato F., Masgras I., Sánchez-Martín C., Rasola A. (2018). Metabolic Plasticity of Tumor Cell Mitochondria. Front. Oncol..

[60] Duggett N.A., Flatters S.J.L. (2017). Characterization of a rat model of bortezomib-induced painful neuropathy. Br. J. Pharmacol..

[61] Stockstill K., Doyle T.M., Yan X., Chen Z., Janes K., Little J.W., Braden K., Lauro F., Giancotti L.A., Harada C.M. (2018). Dysregulation of sphingolipid metabolism contributes to bortezomib-induced neuropathic pain. J. Exp. Med..

[62] Magrangeas F., Kuiper R., Avet-Loiseau H., Gouraud W., Guérin-Charbonnel C., Ferrer L., Aussem A., Elghazel H., Suhard J., Sakissian H. (2016). A Genome-Wide Association Study Identifies a Novel Locus for Bortezomib-Induced Peripheral Neuropathy in European Patients with Multiple Myeloma. Clin. Cancer Res..

[63] Anderson K.C. (2016). Progress and Paradigms in Multiple Myeloma. Clin. Cancer Res..

[64] Robak P., Robak T. (2019). Bortezomib for the Treatment of Hematologic Malignancies: 15 Years Later. Drugs R D.

[65] Pellegrino M., Rizza P., Dona A., Nigro A., Ricci E., Fiorillo M., Perrotta I., Lanzino M., Giordano C., Bonofiglio D. (2019). FoxO3a as a Positive Prognostic Marker and a Therapeutic Target in Tamoxifen-Resistant Breast Cancer. Cancers.

[66] Frattaruolo L., Fiorillo M., Brindisi M., Curcio R., Dolce V., Lacret R., Truman A.W., Sotgia F., Lisanti M.P., Cappello A.R. (2019). Thioalbamide, a thioamidated peptide from amycolatopsis alba, affects tumor growth and stemness by inducing metabolic dysfunction and oxidative stress. Cells.

[67] Armentano B., Curcio R., Brindisi M., Mancuso R., Rago V., Ziccarelli I., Frattaruolo L., Fiorillo M., Dolce V., Gabriele B. (2020). 5-(Carbamoylmethylene)-oxazolidin-2-ones as a promising class of heterocycles inducing apoptosis triggered by increased ros levels and mitochondrial dysfunction in breast and cervical cancer. Biomedicines.

[68] Chim C., Kumar S.K., Orlowski R., Cook G., Richardson P., Gertz M., Giralt S., Mateos M., Leleu X., Anderson K.C. (2018). Management of relapsed and refractory multiple myeloma: Novel agents, antibodies, immunotherapies and beyond. Leukemia.

[69] Sarin V., Yu K., Ferguson I.D., Gugliemini O., Nix M.A., Hann B., Sirota M., Wiita A.P. (2020). Evaluating the efficacy of multiple myeloma cell lines as models for patient tumors via transcriptomic correlation analysis. Leukemia.

