# Supplementary material for: Bortezomib-Loaded Mesoporous Silica Nanoparticles Selectively Alter Metabolism and Induce Death in Multiple Myeloma Cells
Source: Cancers (Basel). 2020 Sep 21;12(9):2709. doi: 10.3390/cancers12092709 (PMC7565423; doi:10.3390/cancers12092709)
Supplement: Supplementary file 1 [file cancers-12-02709-s001.zip › Figure S2-Original WBs images.pptx]

## Slide 1
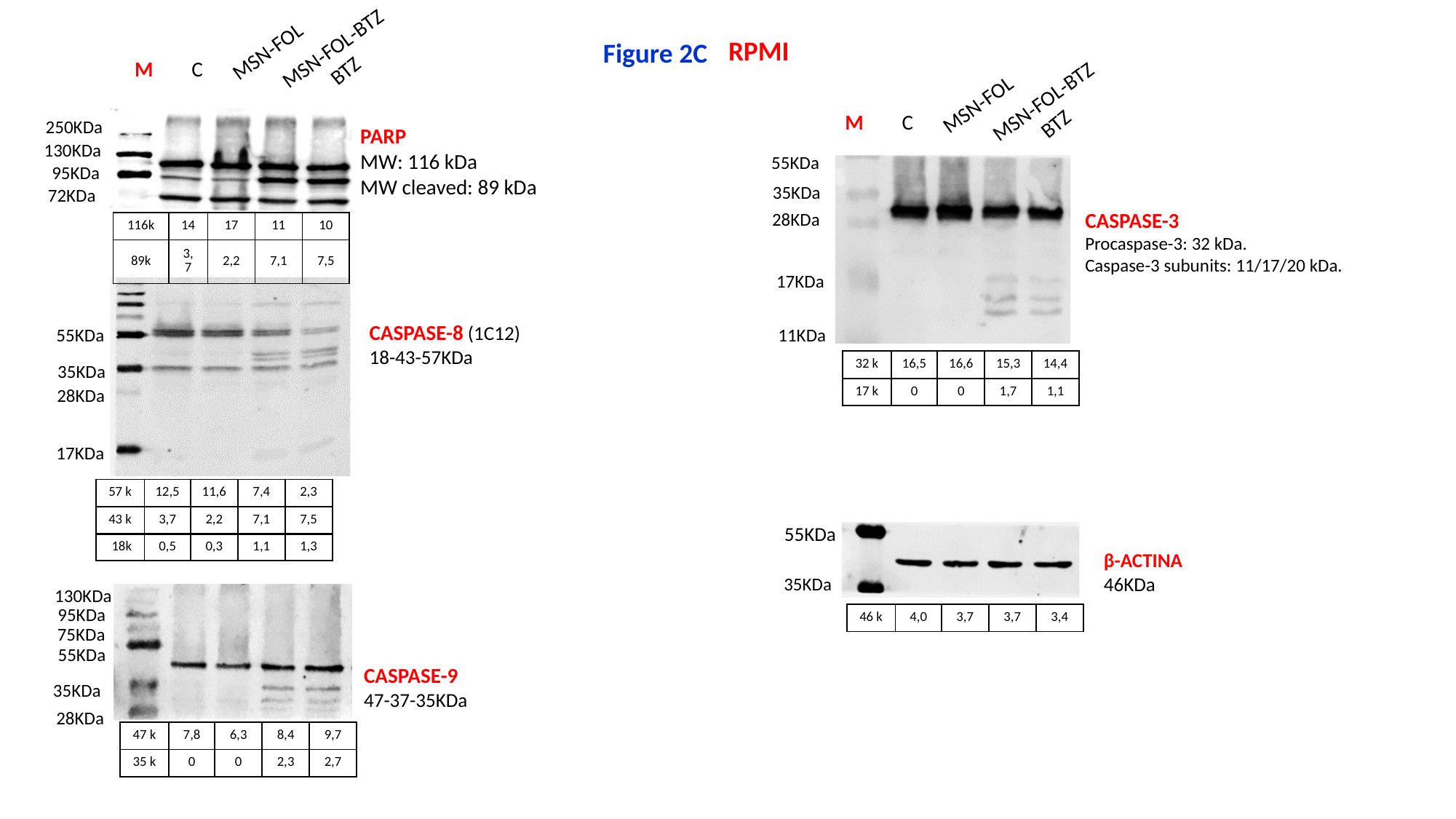

RPMI
MSN-FOL-BTZ
MSN-FOL
C
M
BTZ
Figure 2C
MSN-FOL-BTZ
MSN-FOL
C
M
BTZ
250KDa
130KDa
95KDa
72KDa
PARP
MW: 116 kDa
MW cleaved: 89 kDa
55KDa
35KDa
28KDa
17KDa
11KDa
CASPASE-3
Procaspase-3: 32 kDa.
Caspase-3 subunits: 11/17/20 kDa.
| 116k | 14 | 17 | 11 | 10 |
| --- | --- | --- | --- | --- |
| 89k | 3,7 | 2,2 | 7,1 | 7,5 |
CASPASE-8 (1C12)
18-43-57KDa
55KDa
35KDa
28KDa
17KDa
| 32 k | 16,5 | 16,6 | 15,3 | 14,4 |
| --- | --- | --- | --- | --- |
| 17 k | 0 | 0 | 1,7 | 1,1 |
| 57 k | 12,5 | 11,6 | 7,4 | 2,3 |
| --- | --- | --- | --- | --- |
| 43 k | 3,7 | 2,2 | 7,1 | 7,5 |
55KDa
β-ACTINA
46KDa
35KDa
| 18k | 0,5 | 0,3 | 1,1 | 1,3 |
| --- | --- | --- | --- | --- |
130KDa
95KDa
75KDa
55KDa
35KDa
28KDa
CASPASE-9
47-37-35KDa
| 46 k | 4,0 | 3,7 | 3,7 | 3,4 |
| --- | --- | --- | --- | --- |
| 47 k | 7,8 | 6,3 | 8,4 | 9,7 |
| --- | --- | --- | --- | --- |
| 35 k | 0 | 0 | 2,3 | 2,7 |
55KDa

## Slide 2
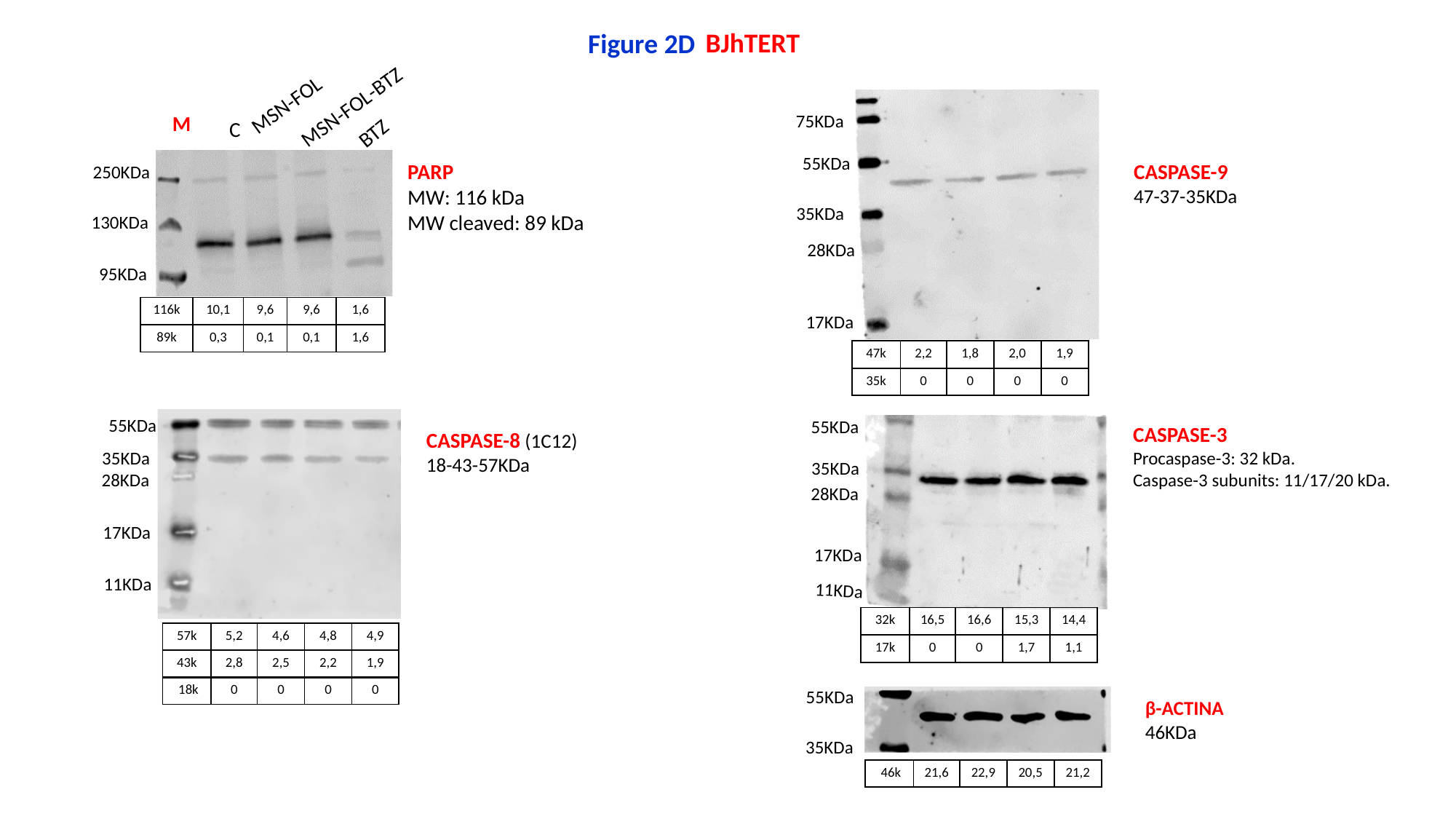

BJhTERT
Figure 2D
MSN-FOL
MSN-FOL-BTZ
75KDa
55KDa
35KDa
28KDa
17KDa
M
C
BTZ
PARP
MW: 116 kDa
MW cleaved: 89 kDa
250KDa
130KDa
95KDa
CASPASE-9
47-37-35KDa
| 116k | 10,1 | 9,6 | 9,6 | 1,6 |
| --- | --- | --- | --- | --- |
| 89k | 0,3 | 0,1 | 0,1 | 1,6 |
| 47k | 2,2 | 1,8 | 2,0 | 1,9 |
| --- | --- | --- | --- | --- |
| 35k | 0 | 0 | 0 | 0 |
55KDa
35KDa
28KDa
17KDa
11KDa
55KDa
35KDa
28KDa
17KDa
11KDa
CASPASE-3
Procaspase-3: 32 kDa.
Caspase-3 subunits: 11/17/20 kDa.
CASPASE-8 (1C12)
18-43-57KDa
| 32k | 16,5 | 16,6 | 15,3 | 14,4 |
| --- | --- | --- | --- | --- |
| 17k | 0 | 0 | 1,7 | 1,1 |
| 57k | 5,2 | 4,6 | 4,8 | 4,9 |
| --- | --- | --- | --- | --- |
| 43k | 2,8 | 2,5 | 2,2 | 1,9 |
| 18k | 0 | 0 | 0 | 0 |
| --- | --- | --- | --- | --- |
55KDa
β-ACTINA
46KDa
35KDa
| 46k | 21,6 | 22,9 | 20,5 | 21,2 |
| --- | --- | --- | --- | --- |

## Slide 3
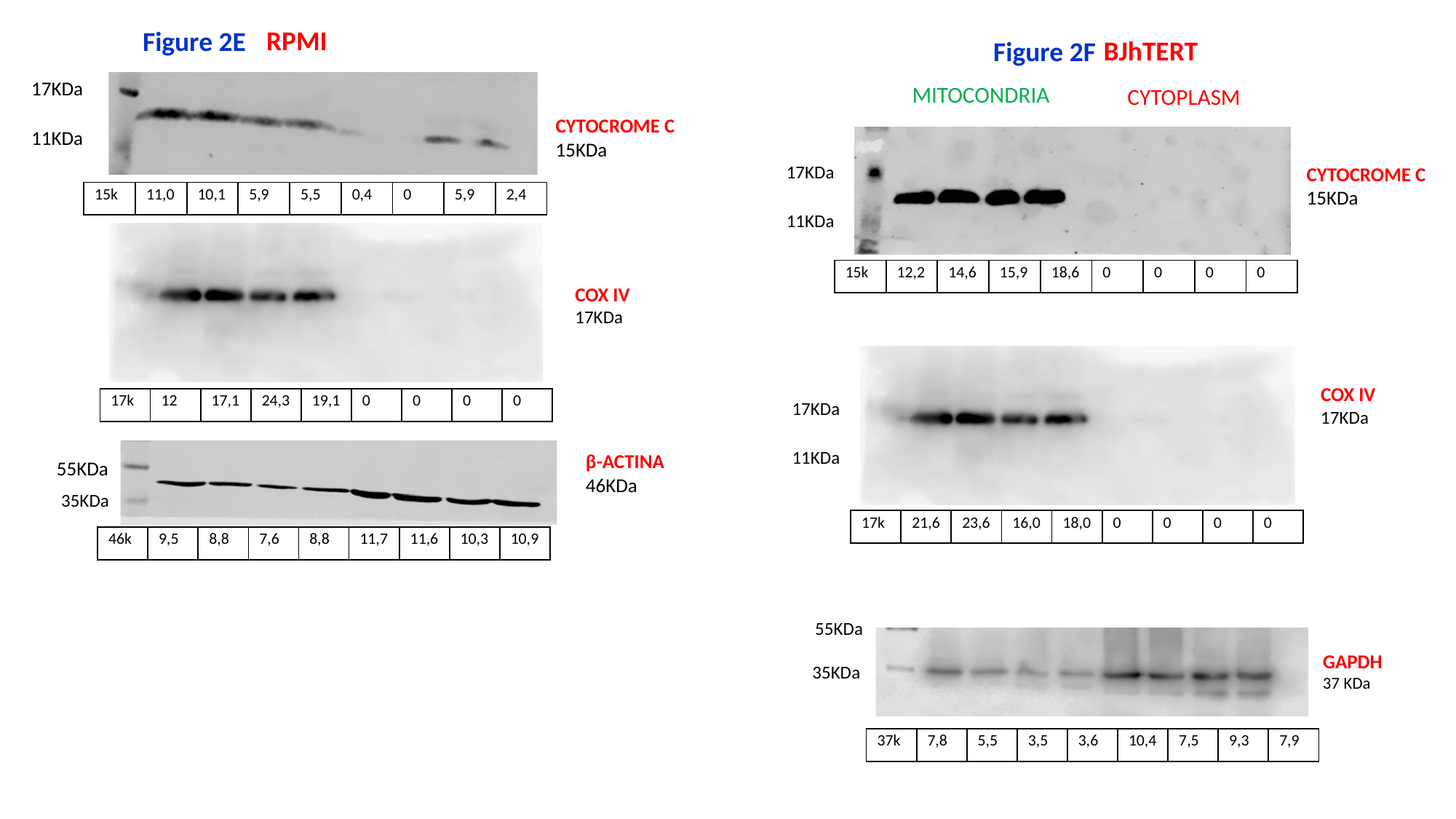

RPMI
Figure 2E
BJhTERT
Figure 2F
17KDa
11KDa
MITOCONDRIA
CYTOPLASM
CYTOCROME C
15KDa
17KDa
11KDa
CYTOCROME C
15KDa
| 15k | 11,0 | 10,1 | 5,9 | 5,5 | 0,4 | 0 | 5,9 | 2,4 |
| --- | --- | --- | --- | --- | --- | --- | --- | --- |
| 15k | 12,2 | 14,6 | 15,9 | 18,6 | 0 | 0 | 0 | 0 |
| --- | --- | --- | --- | --- | --- | --- | --- | --- |
COX IV
17KDa
COX IV
17KDa
| 17k | 12 | 17,1 | 24,3 | 19,1 | 0 | 0 | 0 | 0 |
| --- | --- | --- | --- | --- | --- | --- | --- | --- |
17KDa
11KDa
β-ACTINA
46KDa
55KDa
35KDa
| 17k | 21,6 | 23,6 | 16,0 | 18,0 | 0 | 0 | 0 | 0 |
| --- | --- | --- | --- | --- | --- | --- | --- | --- |
| 46k | 9,5 | 8,8 | 7,6 | 8,8 | 11,7 | 11,6 | 10,3 | 10,9 |
| --- | --- | --- | --- | --- | --- | --- | --- | --- |
55KDa
GAPDH
37 KDa
35KDa
| 37k | 7,8 | 5,5 | 3,5 | 3,6 | 10,4 | 7,5 | 9,3 | 7,9 |
| --- | --- | --- | --- | --- | --- | --- | --- | --- |
55KDa
