# Supplementary figures and images for: Bortezomib-Loaded Mesoporous Silica Nanoparticles Selectively Alter Metabolism and Induce Death in Multiple Myeloma Cells
Source: Cancers (Basel). 2020 Sep 21;12(9):2709. doi: 10.3390/cancers12092709 (PMC7565423; doi:10.3390/cancers12092709)

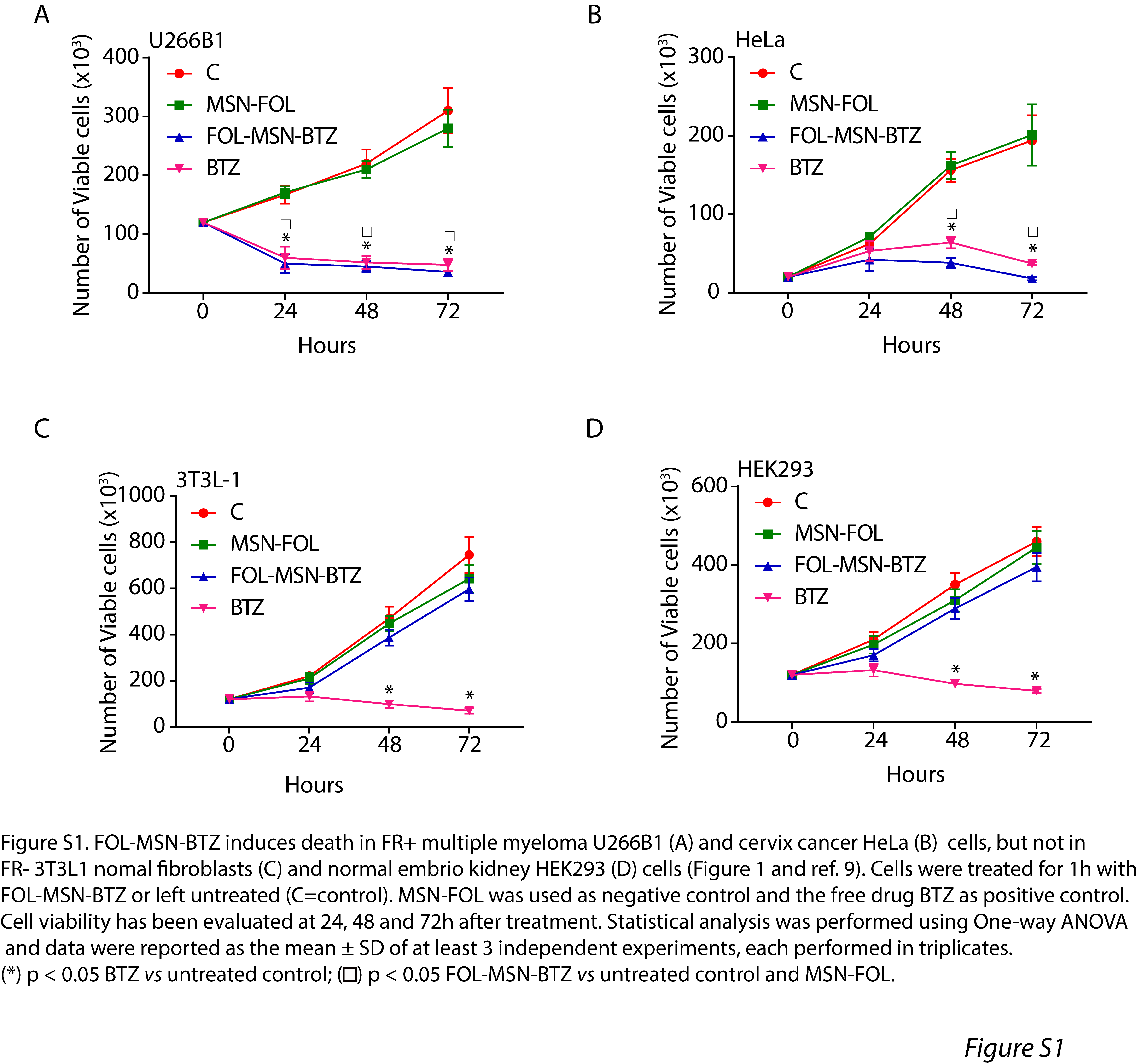

Supplement: Supplementary file 1 [file cancers-12-02709-s001.zip › Fig. S1-Growth various cell lines.tif]
